# "*In their own words*": delineating the contours of dyspnea invisibility in patients with advanced chronic obstructive pulmonary disease from quantitative discourse analysis

**DOI:** 10.1186/s12931-023-02655-4

**Published:** 2024-01-04

**Authors:** Jonathan Dedonder, Christelle Gelgon, Antoine Guerder, Nathalie Nion, Sophie Lavault, Capucine Morélot-Panzini, Jésus Gonzalez-Bermejo, Laelia Benoit, Thomas Similowski, Laure Serresse

**Affiliations:** 1 Institute for the Analysis of Change in Contemporary and Historical Societies (IACS), Université Catholique de Louvain, Louvain-La-Neuve, Belgique; 2 Unité Mobile d’accompagnement et de Soins Palliatifs, AP-HP, Groupe Hospitalier Universitaire APHP-Sorbonne Université, Hôpital Pitié-Salpêtrière, 75013 Paris, France; 3 Service de Médecine de Réadaptation Respiratoire, Département R3S, AP-HP, Groupe Hospitalier Universitaire APHP-Sorbonne Université, Hôpital Pitié-Salpêtrière, 75013 Paris, France; 4 Département R3S, AP-HP, Groupe Hospitalier Universitaire APHP-Sorbonne Université, Hôpital Pitié-Salpêtrière, 75013 Paris, France; 5 Sorbonne Université, INSERM, UMRS1158 Neurophysiologie Respiratoire Expérimentale et Clinique, 75005 Paris, France; 6 Service de Pneumologie, Département R3S, AP-HP, Groupe Hospitalier Universitaire APHP-Sorbonne Université, Hôpital Pitié-Salpêtrière, 75013 Paris, France; 7 Child Study Center, QUALab Qualitative and Mixed Methods Lab, Yale School of Medicine, New Haven, CT USA; 8 Inserm U1018, Team DevPsy, Maison de Solenn, Hôpital Cochin, AP-HP, Paris, France; 9 Fédération “Soins Palliatifs, Accompagnement et Soins de Support”, AP-HP, Groupe Hospitalier Universitaire APHP-Sorbonne Université, Hôpital Pitié-Salpêtrière, 47-83 Bd de l’Hôpital, 75651 Paris Cedex 13, France

**Keywords:** Breathlessness, Dyspnea, Dyspnea invisibility, Chronic obstructive pulmonary disease, Pulmonary rehabilitation, Integrative medicine, Lexicometry

## Abstract

**Background:**

Dyspnea conveys an upsetting or distressing experience of breathing awareness. It heavily weighs on chronic respiratory disease patients, particularly when it persists despite maximal treatment of causative abnormalities. The physical, psychological and social impacts of persistent dyspnea are ill-appreciated by others. This invisibility constitutes a social barrier and impedes access to care. This study aimed to better understand dyspnea invisibility in patients with chronic obstructive pulmonary disease (COPD) through quantitative discourse analysis.

**Methods:**

We conducted a lexicometric analysis (lemmatization, descending hierarchical classification, multicomponent analysis, similarity analysis) of 11 patients' discourses (6 men, severe COPD; immediate postexacerbation rehabilitation) to identify semantic classes and communities, which we then confronted with themes previously identified using interpretative phenomenological analysis (IPA).

**Results:**

Class#1 (*"experience and need for better understanding";* 38.9% of semantic forms, 50% of patients*) *illustrates the gap that patients perceive between their experience and what others see, confirming the importance of dyspnea invisibility in patients' concerns. Class#2 (*"limitations";* 28.7% of forms) and Class#3 (*management";* 13.1% of forms) point to the weight of daily limitations in performing basic activities, of the need to accept or adapt to the constraints of the disease. These three classes matched previously identified IPA-derived themes. Class#4 ("*hospitalization*"; 18.2% of forms) points to the importance of interactions with the hospital, especially during exacerbations, which constitutes novel information.

**Conclusions:**

Lexicometry confirms the importance of dyspnea invisibility as a burden to COPD patients.

**Supplementary Information:**

The online version contains supplementary material available at 10.1186/s12931-023-02655-4.

## Background

Dyspnea is defined as a *'subjective experience of breathing discomfort that consists of qualitatively distinct sensations that vary in intensity*.' It is considered the most severe of the burdens to patients with chronic respiratory diseases [1]. Conveying an upsetting or distressing experience of awareness of breathing, dyspnea is intimately associated with anxiety and fear. Beyond being a symptom, dyspnea is also an existential experience [2, 3]. This becomes particularly true when measures aiming to correct identified etiopathogenic abnormalities fail to provide relief, which defines persistent dyspnea [4, 5]. Persistent dyspnea is associated with diminishing abilities (e.g., inability to walk, talk, leave the house, or get out of bed) and increasing vulnerability, which, together with the development of sentiments of helplessness and unworthiness (e.g., through disappointment about caregivers' responses or embarrassment about symptoms or visible treatments) [6, 7] reduce the patients' spheres of action and thought and lead to social isolation [8].

Persistent dyspnea is charactized by its invisibility, namely the insufficient appreciation by others of what the experience represents for those concerned [2, 3]. Patients with persistent dyspnea complain of poor understanding from others and generally report a lack of solicitude, particularly from healthcare professionals [9]. Dyspnea invisibility leads to injustice by depriving patients of access to adequate care [10, 11], and, more generally, raises human rights issues [12]. To better understand the characteristics and the determinants of the invisibility of persistent dyspnea, we previously conducted a study in patients with severe chronic obstructive pulmonary disease (COPD). This study identified dyspnea invisibility as pleiomorphic, depending on temporality and interlocutors [13] and as an integral part of the patients' lived experience and burden. We reached these conclusions after applying an interpretative phenomenological analysis[14] approach to the content of semistructured patient interviews[13]. Indeed, we identified four themes relating to COPD-related dyspnea in itself: (1) envisioning one's death by suffocation; (2) losing autonomy and hope; (3) coping strategies; and (4) being exhausted by a long-lasting burden. We also identified five themes relevant to dyspnea invisibility: (1) having been fooled by the insidious nature of dyspnea as a warning sign; (2) others' lack of awareness; (3) unshareability of the experience; (4) suffering that cannot be objectively measured; and (5) others' lack of empathetic concern.

Interpretative phenomenological analysis is a hypothesis-free qualitative research method focused on exploring someone's inner world to better understand a particular aspect of human experience [14]. It can be complemented by quantitative discourse analysis for corroboration [15] or to make missed themes emerge. In the present study, ancillary to a previous study [13], we employed advanced lexicometric approaches (such as correspondence analysis and descending hierarchical classification) to analyze the content of the semistructured interviews conducted previously [13] and explore the patients' experience of dyspnea with a different methodology. This lexicometric analysis was conducted independently of the previously described interpretative phenomenological analysis [13]. We then tested the hypothesis that the two methods corroborate and complement each other.

## Methods

### General methodology

The detailed methodology followed to gather the patients' interviews has been described before [13]. In summary, we performed the study in the pulmonary rehabilitation division at the Pitié-Salpêtrière Hospital, a 1600-bed university hospital in Paris, France. Inclusion criteria were (1) age 18 or more; (2) severe COPD (GOLD classification stage 3 or 4 [16]); (3) pulmonary rehabilitation immediately after a severe COPD exacerbation; (4) no known or obvious cognitive deficiencies; and (5) adequate command of French. The study was conducted according to the principles of the Declaration of Helsinki. The participants were informed of the purpose and methods of the study. They provided written consent to participate. The study was approved by the ethical committee of Sorbonne University (*Comité d'Éthique de la Recher*che, decision #19-621). As previously described [13], face-to-face semistructured interviews were conducted to explore the experience of dyspnoea and its perception by others. The interview guide included two questions (*‘How do you live your breathing difficulties?’* ‘*Do you think that people around you realize what you experience with your breathing*?’). Follow-up questions could be asked to gain a deeper understanding of the topic (e.g., ‘*Tell me more about that,*’ ‘*What do you mean by this*’). Over a six month period, we studied 11 consecutive patients with severe COPD (6 men, 5 women; age 71 [64.5–73], forced expiratory volume in 1 s–FEV1–30% [19.5–36.5]) during an immediate post-exacerbation respiratory rehabilitation stay, hence a very homogeneous group of individuals. The characteristics of the patients are provided in Additional file 1: Appendix S1. The median interview duration was 48 min (interquartile range 34.5–51). The interviews were recorded and transcribed verbatim.

### Lexicometric analysis

Quantitative lexicometry consists of a corpus-driven process that deploys computer-assisted linguistic methods and specialized algorithms to exhaustively analyze the content of a given semantic collection. It does so by formally reorganizing text sequences to explore lexicosemantic macrostructures and contrast different parts of the corpus [17]. Lexicometric analysis allows researchers to examine the content of participants' discourse and wording [18], resting upon the notion that recurrences are highly pertinent to identify consistent notions across individuals' discourses. Our lexicometric analysis was conducted by one of the investigators (JD), who specializes in this field of research. The lexical corpus was first expunged from the interviewer's interventions. It was then lemmatized, a process that reduces the different forms of a word to a single one (the "lemma") by removing inflectional differences (e.g., "talks," "talking," and "talked" are reduced to "talk"). The resulting output was automatically divided into units of context based on the number of occurrences and punctuation [19]. Next, matrices crossing individuals and units of context/lemmas (grammatical words such as articles and pronouns excluded) were constituted and explored with descending hierarchical classification [20] and multiple component analysis [19, 21]. Finally, similarity analysis was performed by generating a matrix of the presence or absence of co-occurrences. A network can be built from this co-occurrences matrix, and communities or groups of words can be detected and constitute network modules. Network modularity is a metric that compares the concentration of within-module links with a random distribution of the links between all network nodes. Modularity ranges between -0.5 and 1, and a positive value indicates that the number of within-group links exceeds the number expected in the absence of internal structure. Networks with high modularity (closer to 1) have dense word connections within communities but sparse connections between words in different communities [22]. The UCLouvain community detection algorithm [23] was used in the present study. The analysis was performed using IRaMuTeQ 0.7 alpha 2 R-based software (French dictionary; Laboratoire d'Études et de Recherches Appliquées en Sciences Sociales, Université de Toulouse 3, France).

### Concordance between interpretative phenomenological analysis and lexicometry

For the purpose of triangulation, we combined data analysis triangulation and evaluators triangulation [24–26]. After data collection and transcription, the first evaluator (LS) transmitted the full corpus to the second evaluator (JD). From this point on, a strict non-communication policy between them was followed to avoid any reciprocal influence. LS conducted the IPA analysis, while JD conducted the lexicometric analysis. Upon completion of these two separate processes, each evaluator composed a comprehensive report that was communicated to the other one. The two evaluators then cross-examined their findings, amalgamated the analyses conducted, and formulated interpretations, producing a first version of Table 1 that was subsequently amended and refined after discussion within the research group.

**Table 1:** Correspondence between interpretative phenomenological analysis [13] and quantitative lexicometric analysis

	Interpretative phenomenological analysis – *Themes*	Quantitaive lexicometric analysis – Semantic classes* *(hierarchical descending classification)*	Quantitaive lexicometric analysis – *Semantic communities* *(similarity analysis)*
*Category #1* *Living with COPD-related dyspnea*	*Theme #1* Envisioning one's death by suffocation	Class#1 Experience and need for understanding Class#4 Hospitalization	Community#3 Thing Community#5 Hospitalization Community#6 Feel
	*Theme #2* Losing autonomy and hope	Class#2 Limitations	Community#6 Problem
	*Theme #3* Coping strategies	Class#3 Management	Community#2 Take
	*Theme #4* Being exhausted by a long-lasting burden		Community#3 Thing
*Category #2* *Dyspnea invisibilities*	*Theme #5* Having been fooled by the insidious nature of dyspnea as a warning sign		Community#4 Disease Community#1 Seeing
	*Theme #6* Others' lack of awareness	Class#1 ** Experience and need for understanding	Community#1 Seeing Community#4 Disease
	*Theme #7* An unshareable life experience		Community#7 Feel
	*Theme #8* A suffering that cannot be objectively measured		Community#4 Disease
	*Theme #9* Others' lack of empathetic concern		Community#1 Seeing Community#4 Disease

*words pertaining to dyspnea invisibilities were present in the four semantic classes. ** Class#1 contained words pertinent to the 5 "dyspnea invisibilities" theme. Primary correspondences in black, secondary correspondence in gray

## Results

### Structure of the corpus

A first analysis of the lemmatized corpus showed that one patient's dataset stood out in a self-contained semantic class that did not intersect with the others. The corresponding patient (a native Arabic speaker man) had a correct mastery of French but appeared to use a limited vocabulary and many generic items. This dataset was therefore removed from the initial corpus. The final corpus, from 10 interviews with ten patients, comprised 41,475 words (3488 unique forms or lemmas). It was decomposed into 1157 segments of 35.85 forms on average.

### Hierarchical descending classification

**Fig. 1:**
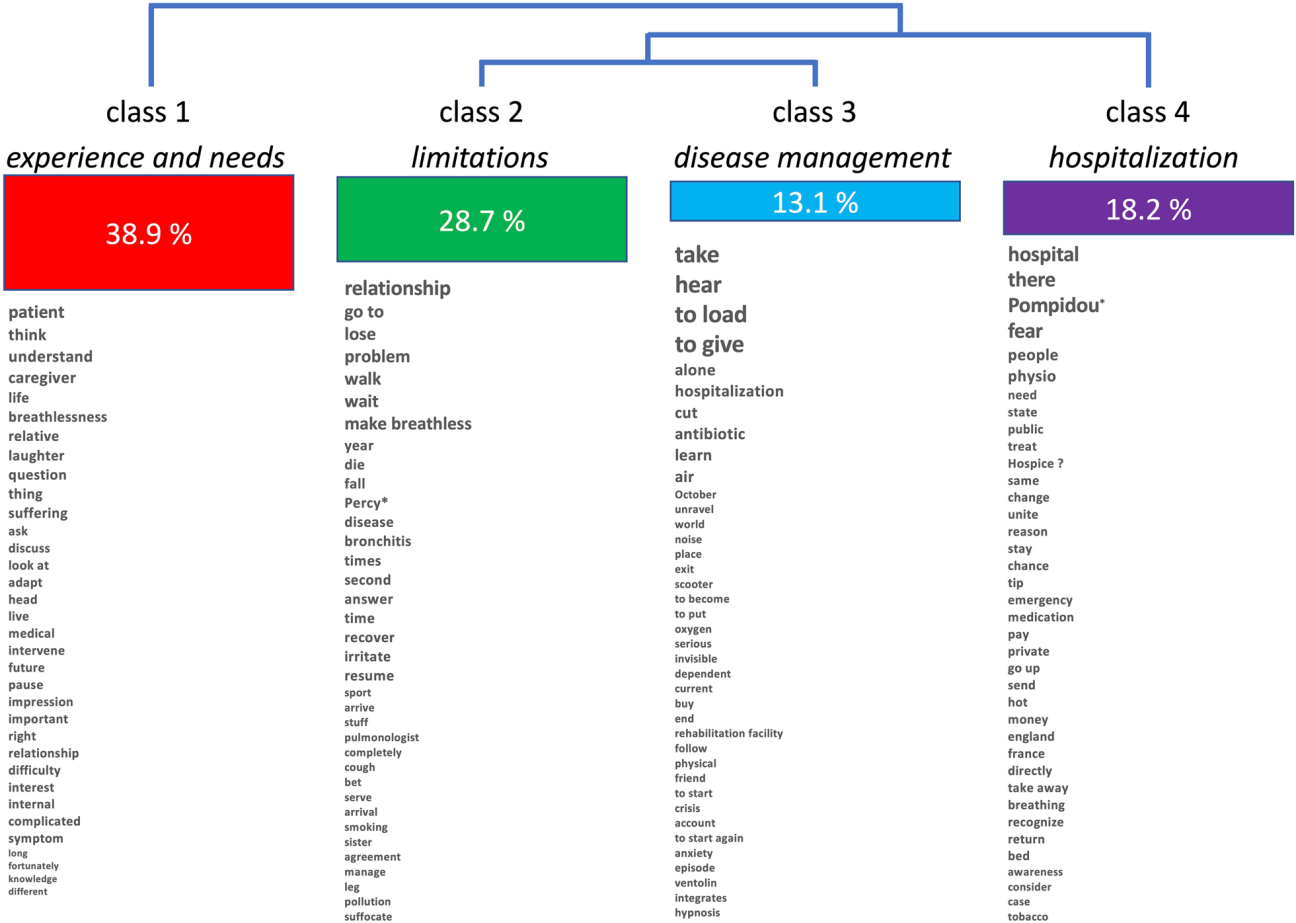
Semantic classes identified by descending hierarchical classification during lexicometric analysis

Hierarchical descending classification allocated 90.92% of the segments (n = 1052) to 4 distinct classes (Fig. 1), as follows.

**Class#1 **("experience and need for understanding"). This class encompasses 38.9% of the classified corpus and is present in half of the patients' discourses. It contains words like "patient," "think," "understand," "life,"; "breathlessness," "suffering. and "caregiver," "question," "talk," and "difficulty". It corresponds to the observation that the patients consistently mention that it is difficult to make others, including the medical profession, aware of their experience and needs (dyspnea invisibility).

**Class#2 **("limitations"). This class comprises 28.7% of the classified corpus and is predominantly driven by two patients. It contains words like "go," "walk," "fall," "lose," "problem," "breathlessness," and "die" that revolve around the daily limitations in performing basic activities and the notion of loss.

**Class#3 **("management"). This class covers 13.1% of the classified corpus. It contains words like "take, –as in "take a drug," or "take charge,"– "antibiotic," and "learn," and focuses mainly on disease management and the importance of taking charge.

**Class#4 **("hospitalization")**.** This semantic class pertains to 18.2% of the corpus segments. It contains words like "hospital" and "there" –as "in the hospital" and "fear." It is centered on the concrete experience of exacerbation-related hospitalization.

### Multicomponent factorial analysis

**Fig. 2:**
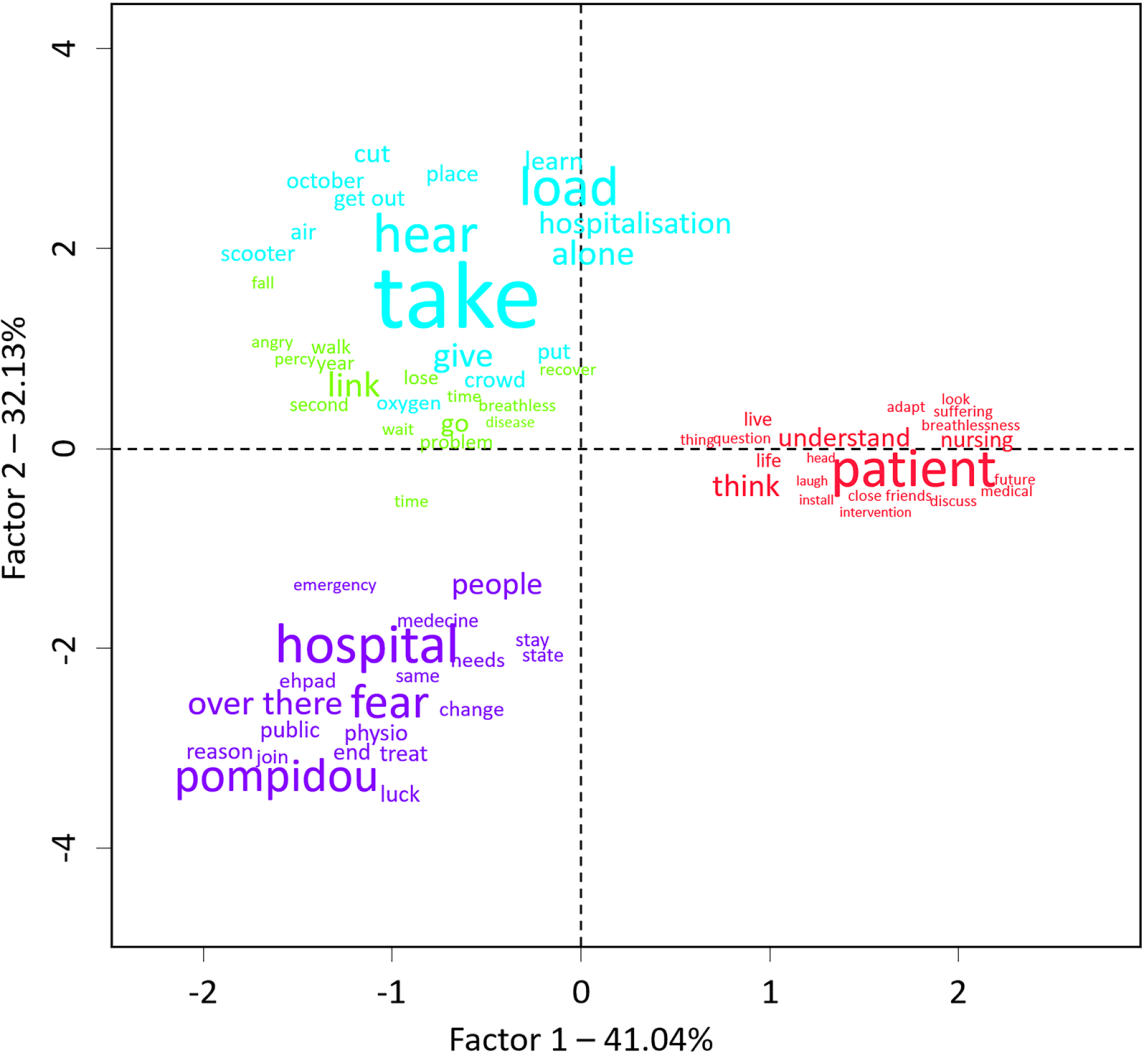
Results of the multiple correspondence analysis following the hierarchical descending classification. Colors are based on the hierarchical descending classification classes. Word sizes are based on the Chi squared association with the class

In multicomponent factorial analysis, the projection of the four semantic classes on two axes explained 73% of the variance of the corpus (41.04% on the horizontal axis, 32.13% on the vertical axis; Fig. 2). On the horizontal axis, Class#1, which relates to "invisibility," is opposed to the three other classes. On the vertical axis (32.13%), Class#2, which relates to "limitations," and Class#3, which relates to "management," largely overlap. In contrast, They appear opposed to Class#4, which relates to "hospitalizations."

### Similarity analysis

**Fig. 3:**
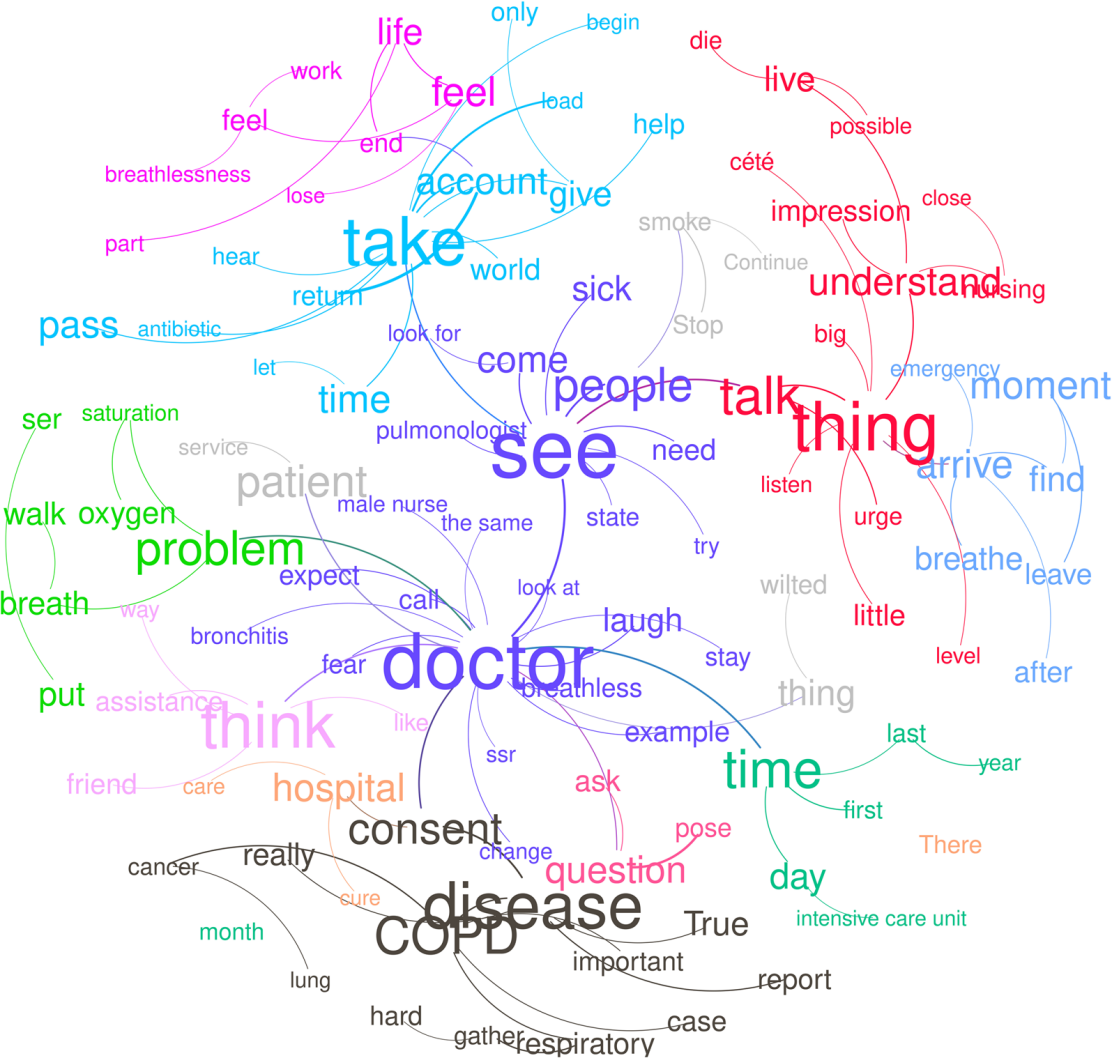
Word cloud of words with frequencies higher than 10. Colors represent semantic communities found from the UCLouvain algorithm. Links between words represent co-occurrences. The sizes of the words were based on the word frequency

Similarity analysis detected 14 semantic communities (Fig. 3), 7 of which represented 72.14% of the corpus. An overall modularity of 0.838 indicated strong connections between within-community terms and weaker relationships between between-community terms. A detailed interpretation appears in Additional file 1: Appendix S2.

### Concordance between lexicometry and interpretative phenomenological analysis

Regarding hierarchical descending classification, we determined that semantic Class#1 contains words relating to the experience of dyspnea (like "breathlessness," "suffering", "difficulty", "symptom," and "future"), which were found to correspond to the IPA Theme#1 "*envisioning death by suffocation*." As mentioned above, this semantic class also contains many words related to dyspnea invisibility. It appears very close to IPA Theme#5 "*other's lack of awareness*," with elements reminiscent of Theme#7 “*an unshareable life experience*” and Theme#8 "*a suffering that cannot be objectively measured*" (Table 1). The meaning conveyed by Class#1 is exemplified in sentences like "…*Why is empathy greater among patients than between patients and healthcare providers? It's because there's a recognition of each other's suffering…*" (LLL, female), or "…*Moreover, some patients have trouble breathing without having desaturations […] it's terrible to think that a healthcare provider believes […][the] numbers rather than the person*…" (DER, female). Regarding Class#2, the IPA-lexicometry confrontation led to the conclusion that the semantic content of the class corresponded to IPA Theme#2 "*losing autonomy and hope*" (Table 1). Indeed, most of the words in this class correspond to either an action ("walking"), a difficulty to perform an action, or the realization of a loss, as exemplified by sentences like "…*I had gone to see my doctor because I was experiencing difficulties in breathing […] his office […]wasn't very far. When I arrived, I was really out of breath…*" (CHA, female) or "…*I used to think it was just recurring bronchitis. I didn't realize it was going to be a permanent condition…*" (CHM, male). The content of Class#3 has a strongly therapeutic and educational connotation, which is consistent with the rehabilitation context of the study, and which covers IPA Theme#3 "*coping strategies*". This is illustrated by sentences like "… *So, with all […] the precautions that need to be taken and the fact that you should take antibiotics as soon as you have an infection…*" (RSH, female) or "*… you have to be careful, you have to avoid things you shouldn't do […] because it will make [you] breathless*…" (COR, male). In contrast, the content of semantic Class#4, centered on hospital stays, was not identified as a main theme by the IPA.

Regarding similarity analysis, we found that four of the seven main semantic communities were found to relate to the experience of living with dyspnea, generally corresponding to Themes#1–4 of the IPA. Likewise, the "take" community (13.5% of the corpus) involved the terms "care," "management," "self-care," and "learning," which is very close to the notions expressed in IPA Theme#3, "*management and coping strategies*." The "thing," "feel," and "hospitalization" communities included the terms “breathing,” “living,” and “dying,” all determinants of the concerns identified by IPA Theme#1 "*envisioning one's death by suffocation*," and IPA Theme#4 "*being exhausted by a long-lasting burden"*, both themes that highlight the impact of dyspnea on daily life and on expectancies. This is exemplified by sentences like “…*It is as if you died many little deaths. Every time you suffocate, you wonder if you are going to die […] COPD patients are exhausted from almost dying so many times*…” (DER, female). The same was true for the "problem" community (8.3% of the corpus), with words like "walk," "oxygen," "move," and "handicap." Of note, the "time" community (8.4% of the corpus) concentrated on stories of hospitalizations, with emphasis on words like "first," "intensive care unit," and "hospitalizations." As in the case of semantic Class#4, centered on hospital stays, this particular preoccupation did not emerge as a main theme in the IPA process.

The other three main semantic communities ("seeing," "disease," and "feeling") were found to revolve around the notion of dyspnea invisibility and to correspond to IPA Themes#5–9 (Table 1). The "seeing" community (20.5% of the corpus) refers to the need to be recognized as being sick at an individual level. The "disease" community (9.4% of the corpus) more closely relates to the invisibility of COPD at a societal level. The "feeling" community (6.5% of the corpus) corresponds to both the experience of dyspnea (IPA Theme #1) and its invisibility through unshareability IPA Theme#7). Of note, the "thing" community (11.95% of the corpus), with words like "talk," "understanding," "feeling," "live," and "death," was found to relate with both dyspnea experience (IPA Theme#1) and dyspnea invisibility in general.

## Discussion

### Summary of findings

This study shows that a lexicometric analysis of the discourse of patients with severe COPD undergoing post-exacerbation rehabilitation corroborates the results of the interpretative phenomenological analysis previously performed on the same corpus [13], by pointing to the importance of the burden of persistent dyspnea, to the importance of dyspnea invisibility, and to the pleiomorphic nature of this invisibility. In addition, the study shows that hospitalizations represent a specific patient concern.

### Interpretation of the comparison between lexicometric and qualitative analyses [13]

As described in the corresponding section of "Results" and summarized in Table 1, there appeared to be a good coherence between the semantic classes and the semantic communities identified by the lexicometric analysis and the IPA-identified themes. The lexicometric analysis confirmed the dominance of the experience of dyspnea and dyspnea invisibility among patients' preoccupations. The large overlap shown by multicomponent factorial analysis between semantic Class#1 and semantic Class#2 (Fig. 2) suggests concomitant discourses and possibly the difficulty of differentiating between the description of a problem and its resolution. In other words, when talking about disease-related difficulties, patients also discuss coping means. Of note, the multicomponent analysis clearly shows (Fig. 2) that semantic Class#1 has a particular position, being completely separated from the other three classes. Insofar as the triangulation process showed that Class#1 was closely related to dyspnea invisibility IPA themes, this finding brings additional justification to the identification of dyspnea invisibility as an overarching worry for concerned patients. Also of note, the "*a suffering that cannot be objectively measured*" IPA theme was not well identified by the descending hierarchical classification and the similarity analysis, which represent the only significant divergence that we observed between the lexicometric approach and the interpretative phenomenological analysis approaches [12]. Finally on this, it is important to emphasize that the lexicometric analysis identified hospital stays as having a specific place in the patient's discourse. The corresponding semantic class (Class#4) was not found to correspond to any of the IPA themes. In multicomponent analysis, Class#4 appears distinct from Class#2 and Class#3 (Fig. 2), indicating that the hospitalization experience has a specific place in the patient's discourse. This finding highlights the interest in combining distinct methodologies when analyzing patients' discourses.

### Position of the study in the existing literature

The notion of dyspnea invisibility was put forward by Gysels and Higginson in 2006 [10] who conducted a qualitative study in 18 COPD patients suffering from breathlessness and concluded that low access to services was related to dyspnea being unrecognized, stigmatized, and discredited. The concept has been extended to chronic respiratory diseases in general [2, 3], with recent data showing that interactions between patients and caregivers are abnormally rare during clinical consultations [11]. In recent years, qualitative studies have helped better describe what dyspnea represents for COPD patients (e.g., [6, 27–29]) and confirmed the reality and importance of its invisibility [30], which proceeds from multiple mechanisms [13]. In this context, the present study appears to be the first to use advanced lexicometric analysis in the field of COPD. This approach has been used to address different issues (e.g., the experience of families of people with diabetes [31]; fathers' role in supporting breastfeeding in preterm infants [32]; women's participation in human immunodeficiency virus clinical trials [33]; patients' experience after the onset of dialysis [15], etc.), but we have been unable to find examples of its application to respiratory medicine. Triangulation between lexicometry and thematic analysis has been used before in various domains including educational science, communication, political science, and medicine (eg. in dialysis patients [15], but the combination with IPA that we used to enhance the reliability and validity of our conclusions is original.

### Study limitations

The size of our study population (n = 10) is small in comparison to other lexicometric studies that we identified as similar to ours and that included from 17 to 100 participants [15, 31, 33–38]. This prevented us from testing statistical associations between semantic outputs and patients' characteristics. However, the size of our corpus (41,475 words and 3488 unique forms) compares with others, and more than 90% of it was allocated to four semantic classes. This figure is very high: Montalescot et al. [15], in dialysis patients considered their 84% such repartition "very satisfying" compared with the 60–75% reported in other studies. We believe that this reflects the very homogeneous nature of our study population, but we acknowledge that this limits the generalizability of our results. This is all the more so that, for pragmatic reasons, we studied COPD patients having reached an advanced of the disease, and in the particular context of a post-exacerbation rehabilitation program. We also recognize that because of the exclusion of one patient due to limited vocabulary, the corpus used for the lexicometric analysis is not the same as the corpus used for the previous interpretative phenomenological analysis. We also acknowledge that we did not formally assess the semantic language abilities of our patients. However language dysfunction has been identified in COPD patients and correlated with hypoxemia [39], which could have interfered with our results.

### General interpretation and clinical implications

This study confirms the importance of dyspnea invisibility for patients with severe COPD. It reinforces the notion that dyspnea invisibility proceeds from distinct mechanisms and indicates that dyspnea invisibility represents an overarching existential concern for these patients. It also points to hospitalizations as a specific patient concern, something the previously conducted interpretative phenomenological analysis had not readily identified [13]. This finding is coherent with the relationship formerly established between COPD exacerbations and the prevalence of post-traumatic stress symptoms in this population [40].

Insofar as dyspnea invisibility represents a barrier between patients and adequate care [10, 30, 41] and a social injustice, our results provide an additional incentive to find corrective measures. Recent data indicate that it is difficult to initiate a dialog about dyspnea during medical interactions [11]. One possible way to address this difficulty could be, hypothetically, to develop and test a specific "dyspnea invisibility" questionnaire that would aim to alert caregivers about patients' perceived lack of attention to their experience of dyspnea. Indeed, recent data suggest that prior knowledge of patients' answers to medical questionnaires can influence physicians' approaches to problem solicitation in certain contexts [42]. Such a questionnaire would more likely to be effective if using the patients' own words [43], as they are identified by our study.

## Conclusion

The present linguistic study emphasizes the importance of dyspnea invisibility for patients concerned by persistent breathlessness.

### Supplementary Information

**Additional file 1: Appendix S1.** Characteristics of the 11 interviewed patients. Tabular data providing a detailed description of patients' characteristics and clinical history.Reproduced with permission from Serresse L, Guerder A, Dedonder J, Nion N, Lavault S, Morelot-Panzini C, Gonzalez-Bermejo J, Benoit L, Similowski T. 'You can't feel what we feel': Multifaceted dyspnoea invisibility in advanced chronic obstructive pulmonary disease examined through interpretative phenomenological analysis. Palliat Med 2022: 36: 1364–1373. **Appendix S2.** Detailed interpretation of the similarity analysis. Detailed description of the semantic communities identified during the similary analysis lexicometric process.

## Data Availability

All data will be made available by the authors upon reasonable request.

## Abbreviations

COPD: Chronic obstructive pulmonary disease
FEV1: Forced expiratory volume in one second
IPA: Interpretative phenomenological analysis
