# Supplementary material for: "In their own words": delineating the contours of dyspnea invisibility in patients with advanced chronic obstructive pulmonary disease from quantitative discourse analysis
Source: Respir Res. 2024 Jan 4;25:21. doi: 10.1186/s12931-023-02655-4 (PMC10768212; doi:10.1186/s12931-023-02655-4)
Supplement: Supplementary file 1 — Additional file 1: Appendix S1. Characteristics of the 11 interviewed patients. Tabular data providing a detailed description of patients' characteristics and clinical history.Reproduced with permission from Serresse L, Guerder A, Dedonder J, Nion N, Lavault S, Morelot-Panzini C, Gonzalez-Bermejo J, Benoit L, Similowski T. 'You can't feel what we feel': Multifaceted dyspnoea invisibility in advanced chronic obstructive pulmonary disease examined through interpretative phenomenological analysis. Palliat Med 2022: 36: 1364–1373. Appendix S2. Detailed interpretation of the similarity analysis. Detailed description of the semantic communities identified during the similary analysis lexicometric process. [file 12931_2023_2655_MOESM1_ESM.docx]

**Additional file 1**

**"*In their own words*": delineating the contours of dyspnea invisibility in patients with advanced chronic obstructive pulmonary disease from quantitative discourse analysis.**

**Appendix S1.** Characteristics of the 11 interviewed patients.

| **gender**  *number* | 6 men / 5 women |
| --- | --- |
| **age** (years)  *median [interquartile range]* | 71  [64,5-73] |
| **body mass index** (kg.m^-2^)  *median [interquartile range]* | 21,8  [16,1-27,5] |
| **cumulative tobacco consumption** (pack-year)  *median [interquartile range]* | 45  [40-65] |
| **FEV1** (% pred)  *median [interquartile range]* | 30  [19,5-36,5] |
| **FVC** (% pred)  *median [interquartile range]* | 68  [64-93,5] |
| **FEV1/FVC** (%)  *median [interquartile range]* | 30  [27-42] |
| **GOLD** I-II-III-IV stage | 0 / 1/ 4 / 6 |
| **GOLD** A-B-C-D stage | 0 / 3 / 1 / 8 |
| **Comorbidities** |  |
| At least one | 10 |
| Obesity | 2 |
| Diabetes | 1 |
| Hypertension | 6 |
| Ischemic heart disease | 3 |
| Cardiac insufficiency | 1 |
| Obstructive sleep apneas | 1 |
| Charlson comorbidity index | 4  [3,5-5] |
| **History of exacerbations** |  |
| number during the past two years | 4  [2,5-5] |
| ICU admission in the past (y/n)) | 8 |

| **History of the exacerbation preceding rehabilitation facility admission** |  |
| --- | --- |
| Duration of hospitalisation prior to rehabilitation facility admission (days)  *median [interquartile range]* | 14  [8-21] |
| Non-invasive ventilation during this hospitalisation (y/n) | 7/4 |
| ICU stay during this hospitalisation (y/n) | 5/6 |
| if yes: with non-invasive ventilation (y/n) | 5/6 |
| if yes: with intubation (y/n) | 1/10 |
| **Status at the time of interview** |  |
| Delay between interview and rehabilitation facility admission (days)  *median [interquartile range]* | 11  [9-24] |
| **Treatment** |  |
| LABD alone | 2 |
| LABD+ICS | 8 |
| supplemental oxygen (permanent) | 7 |
| supplemental oxygen (intermittent) | 2 |
| non-invasive ventilation (nocturnal) | 6 |
| non-invasive ventilation (nocturnal+exercise) | 0 |
| 6 minutes walking distance (m) | 165  [116-215] |
| VQ11 quality of life questionnaire * | 43  [36,75-43,5] |

FEV1: forced expiratory volume in 1 s

FVC: forced vital capacity

LABD: long-acting bronchodilators (any class; single or combined)

ICS: inhaled corticosteroids

ICU: intensive care unit

* the VQ11 is an 11-item health-related quality of life questionnaire specific to chronic obstructive pulmonary disease ^41^, scored from 11 (no impact of COPD on quality of life) to 55 (worst impact). A VQ11 score ≥ 22 indicates significantly altered COPD-related alteration in quality of live.

**Reproduced with permission from**

Serresse L, Guerder A, Dedonder J, Nion N, Lavault S, Morelot-Panzini C, Gonzalez-Bermejo J, Benoit L, Similowski T. 'You can't feel what we feel': Multifaceted dyspnoea invisibility in advanced chronic obstructive pulmonary disease examined through interpretative phenomenological analysis. *Palliat Med* 2022: 36: 1364-1373.

**Appendix S2.** Detailed interpretation of the similarity analysis.

Similarity analysis detected 15 semantic communities, with overall modularity of 0.838 indicating strong connections between within-community terms and weaker connections between between-community terms. Seven communities represented 72.14% of the corpus.

The largest community (20.53%) is concentrated around the notion of "seeing" ("voir" in French). It also involves terms as "doctor", "people", "need",. Within this community, the importance of social support by "seeing" other "people" emerges. The association between "need" and "see", refers to the importance of being perceived as sick and to make dyspnea known. The constructs around the notion of the doctor point to the importance of the patient-doctor relationship. This relationship must be positive and it is important to evoke all the problems experienced. This community thus clearly revolves around the notion of visibility/invisibility to others (what the patients say and want, how do the interlocutors react and what do they provide).

The second community (13.50%) is concentrated around the notion of "taking" ("prendre" in French). Above all, this community refers to the notion of "care", "management", and their impact on concrete aspects of life. It comprises constructions such as taking antibiotics or gaining weight. It also comprises logics such as "learning" to "take" charge of oneself "alone", taking one's "time" to carry out daily activities that are made more complex by the reality of the illness. This community therefore refers to the concrete experience, but also to the resilience of patients and their ability to take charge of themselves, to adapt life agency to disease-related limitations, and to find a rhythm.

The third community (11.95%) is articulated around the generic term "thing" ("chose" in French), which is associated with: “to talk”, "to breathe", "to arrive", "to understand", "impressions", "to live", "to die". This community therefore evokes the experience of patients with persistent dyspnea, this "thing" that "happens" to them and disrupts the way they "live" by giving the "impression" of "dying" (dyspnea as a thing that happens to you and changes life through a permanent feeling of impending death). The association between the terms "breathing", "living", and "dying" reflects the very strong link between breathing and life. This corresponds to the "breathing symbolism and perspective of death" theme identified by the interpretative phenomenological analysis. This importance of the recognition of the disease is also found in "talk". Talking serves not only as a social support but also as a vector of knowledge and recognition of the experience.

The fourth community (9.42%), centred on "disease" ("maladie" in French) is the one that corresponds to the patients' desire to "know" the "disease" and have it known by "the doctors" and "the society". Here, the "invisible" character of the symptoms of "COPD" is mentioned several times. Comparisons with "Cancer" that is more visible and known to the general public are frequent. This is also the community where the importance of an early "diagnosis" of the disease is mentioned.

The fifth main community (8.44%) is built around the term “time” and the stories of "hospitalisations" ("hospitalisation" in French) and more specifically around that of the first "time". It includes concepts such as the patient’s stay in "reanimation" (intensive care). How they experienced the life in the “intensive care unit”.

The sixth main community (8.3%) is around the term “problem” and reflect their difficulties performing everyday tasks, like “Walking” or “moving”. This community also reflect their relation and dependance with the oxygen supplies.

Finally the seventh semantic community (6.47%), the "Feel" ("sentir" in French) is related to dyspnea experience in the phenomenological way (the intimate, experiential or even existential experience). This community is associated with suffering (pain/dyspnea, life/death). It refers to dyspnea invisibility by nature, which will remain and so cannot be shared.
